# Examining health facility financing in Kenya in the context of devolution

**DOI:** 10.1186/s12913-021-07123-7

**Published:** 2021-10-13

**Authors:** Angela Kairu, Stacey Orangi, Boniface Mbuthia, Joanne Ondera, Nirmala Ravishankar, Edwine Barasa

**Affiliations:** 1 Health Economics Research Unit (HERU), KEMRI-Wellcome Trust Research Program, P.O. Box 43640, – 00100, Lenana Road, Nairobi, Kenya; 2 ThinkWell Kenya, P.O. Box 52201 –, Nairobi, 00100 Kenya; 3 Independent Consultant, P.O. Box 102370-00101, Nairobi, Kenya; 4 ThinkWell, 1701 Rhode Island Ave NW, Washington, DC 20036 USA; 5 Centre for Tropical Medicine and Global Health, Nuffield Department of Medicine, University of Oxford, Peter Medawar Building for Pathogen Research, South Parks Road, Oxford, OX1 3SY UK

**Keywords:** Public finance management, Funds flow, Devolution, Kenya

## Abstract

**Background:**

How health facilities are financed affects their performance and health system goals. We examined how health facilities in the public sector are financed in Kenya, within the context of a devolved health system.

**Methods:**

We carried out a cross-sectional study in five purposely selected counties in Kenya, using a mixed methods approach. We collected data using document reviews and in-depth interviews (no = 20). In each county, we interviewed county department of health managers and health facility managers from two and one purposely selected public hospitals and health center respectively. We analyzed qualitive data using thematic analysis and conducted descriptive analysis of quantitative data.

**Results:**

*Planning and budgeting***:** Planning and budgeting processes by hospitals and health centers were not standardized across counties. Budgets were not transparent and credible, but rather were regarded as “wish lists” since they did not translate to actual resources. *Sources of funds***:** Public hospitals relied on user fees, while health centers relied on donor funds as their main sources of funding. *Funding flows***:** Hospitals in four of the five study counties had no financial autonomy. Health centers in all study counties had financial autonomy. Flow of funds to hospitals and health centers in all study counties was characterized by unpredictability of amounts and timing. Health facility expenditure: Staff salaries accounted for over 80% of health facility expenditure. This crowded out other expenditure and led to frequent stock outs of essential health commodities.

**Conclusion:**

The national and county government should consider improving health facility financing in Kenya by 1) standardizing budgeting and planning processes, 2) transitioning public facility financing away from a reliance on user fees and donor funding 3) reforming public finance management laws and carry out political engagement to facilitate direct facility financing and financial autonomy of public hospitals, and 4) assess health facility resource needs to guide appropriate levels resource allocation.

**Supplementary Information:**

The online version contains supplementary material available at 10.1186/s12913-021-07123-7.

## Introduction

Public healthcare facilities are not only a major avenue for the delivery of key healthcare interventions, but also consume a substantial amount of health sector resources. For instance, public hospitals are said to consume between 30 to 50% of health sector budgets in low-and-middle income countries [1]. A key determinant of the performance of public health facilities is the healthcare purchasing arrangements, and specifically the mechanisms used to finance their operations. How public healthcare facilities are financed can affect health system goals in several ways. For example, the reliability of sources of funds may influence achievement of financial risk protection goals. Under-resourced healthcare facilities are likely to deliver poor quality services and outcomes of care. Resource allocation mechanisms for public healthcare facility resources may influence the efficiency and equity of their operations, as well as affect the quality of services provided. Payment mechanisms, the efficiency of their disbursements, and the autonomy healthcare facilities have over their finances may generate unintended incentives to healthcare providers as well as compromise the operational efficiencies of healthcare facilities. Understanding how public healthcare facilities are financed is therefore an important research question.

Financing arrangements for public facilities changed dramatically in Kenya in 2013, when the country transitioned from a centralized to a devolved system of government characterized by a two-tier government i.e. national government and 47 sub-national authorities known as counties [2]. Under this devolved governance arrangement, the national government retained policy formulation and regulation roles while the service delivery function was transferred to counties. Public sector health service delivery is organized into four levels: (a) community services- level 1 community units providing community-based demand creation activities, (b) primary health services- (level 2 dispensaries and level 3 - health centers), (c) county referral services (level 4 and 5 hospitals) and (d) national referral services (level 6 hospitals). The county authorities are responsible for providing services in levels (a) to (c) and the national government is responsible for providing national referral services [3].

Since independence, Kenya has had several health financing reforms in the health sector that have affected public health facility financing. The health sector was predominantly tax funded until 1989, when the country introduced user fees in public hospitals and peripheral health facilities (health centers and dispensaries) that offer outpatient primary healthcare services [4, 5]. In 2004, the Kenyan government abolished user fees in public health centers and dispensaries, except for a flat registration fee of Kenyan shillings (KES) 10 (USD 0.1) dispensaries and KES 20 (USD 0.2) in health centers [6]. Public hospitals were, however, allowed to continue collecting user fees under a cost-sharing arrangement where hospitals received partial supply side subsidies from the central government and charged fees to users of healthcare services. Public hospitals operated bank accounts where their financial resources from user fees, supply side subsidies (drugs and supplies, support for operations and maintenance and staff costs) and National Hospital Insurance Fund (NHIF) reimbursements were collected, and had financial autonomy to manage and use these funds [7]. Hospitals set up health facility management committees (HFMCs), that comprised of hospital leadership and community representatives, to oversee the management of hospital resources.

In contrast, health centers and dispensaries faced funding flow challenges because centrally held funds meant for their operations often failed to reach these facilities [8]. For instance, an assessment found that almost a third of allocations approved were not received by these health facilities in the mid-2000s [8]. To address this funds flow challenge at the primary healthcare facility level, the government established the health sector services fund (HSSF) in 2010 to reform funding flow arrangements of primary healthcare facilities by facilitating the direct transfer of funds to bank accounts operated by health centers and dispensaries, the Ministry of Public Health and Medical Services further issued legal notice 79 (2007) and 155 (2009) that allowed revenues for health facilities not to be transferred to the consolidated fund account at the District Treasury, and setting up health facility committees (HFCs), comprised of facility leadership and community representatives, to oversee the management of these funds at the facility level [8]. HSSF was financed by the government, and development partners (the World Bank and the Danish International Development Agency (DANIDA) [8]. This reform ensured that primary healthcare facilities had the financial autonomy needed to operate effectively.

In 2013, just as the transition to a devolved system of government was commencing, the newly elected national government abolished user fees completely in public health centers and dispensaries. The government set up a user fee reimbursement fund to replace user fees forgone and structured this as conditional grant to county governments, ringfenced for use by primary healthcare facilities. Public hospitals continued to charge user fees. The country also implemented a new public finance management (PFM) law (PFM act 2012) that among others set up a centralized county revenue fund (CRF), where all county revenues are to be operated from [9]. The centralization of county financial management usurped financial autonomy from public hospitals [10]. In 2014, a special purpose account (SPA) was introduced to channel conditional grants from donors and government. The SPA facilitated the ringfencing of donor and government funds for specific use, in contrast to the CRF which was for general use.

Alongside the user fee removal policy in 2013, the government also introduced a free maternity policy that was managed by the Ministry of Health (MOH) and used a case based payments to reimburse public hospitals and health centers for maternal health services, specifically deliveries [11]. The management of the free maternity policy was transferred to the NHIF in 2017, rebranded as Linda Mama (Swahili for take care of the mother) programme, and expanded to include antenatal care, post-natal care and delivery-related complications at this time. In 2015, the NHIF also expanded the benefit package for the supa-cover insurance scheme to include outpatient care and introduced capitation as a mechanism for paying health facilities for these services [12]. This introduced new payments from NHIF to health centers, while previously NHIF payments were largely flowing to hospitals. However, whether public facilities can retain and spend these funds is determined by county government policies.

Anecdotal evidence suggests that the evolution of health financing policies as well as the shift to devolution has introduced significant inter-county variation in facility financing arrangements. Against this backdrop, we conducted a study using a mixed methods approach to assess the financing of county public healthcare facilities in Kenya within the context of devolution.

## Methods

### Conceptual framework

**Fig. 1:**
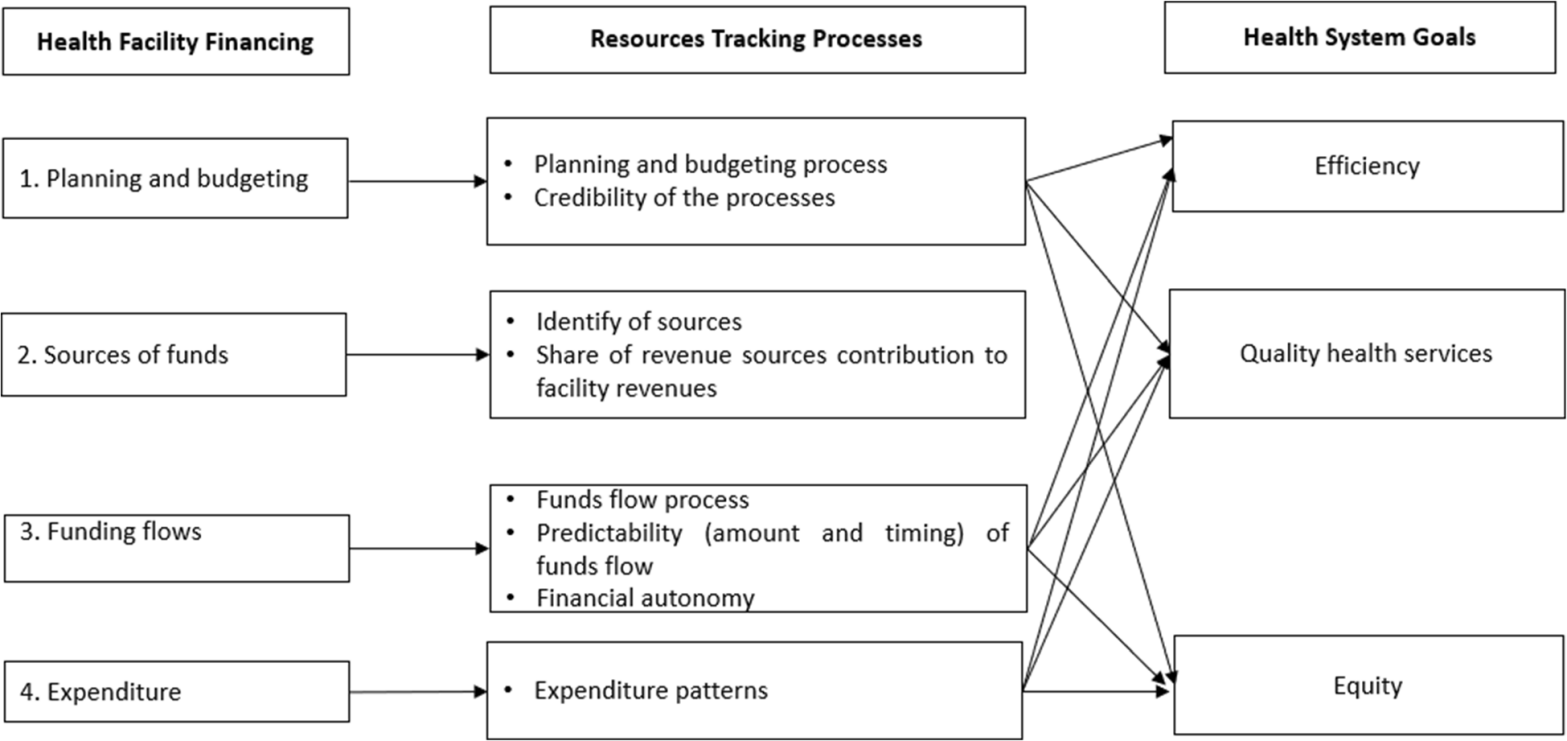
Conceptual framework

Figure 1 outlines the study’s conceptual framework. The framework unpacks health financing into four domains namely planning and budgeting, sources of funds, flow of funds to health facilities, and the spending of these funds at the facility level. The framework identifies aspects of these domains that may influence health systems goals (efficiency, equity, and quality of care). The framework domains and aspects of the domains guided the tools development, data collection and analysis in this study.

### Study design

We conducted a multiple-methods cross-sectional study where we collected both qualitative and quantitative data. This approach allows for comprehensive understanding of data from each method, and complements and validates the data obtained from both methods [13]. All methods were performed in accordance with relevant guidelines and regulations.

### Study sites

We purposively sampled five counties (Table 1), guided by negotiations with the MOH and council of governors to reflect convenience, geographical variation, and variation in health resource indicators. We have anonymized the counties to maintain confidentiality of the study participants.

**Table 1:** Characteristics of study counties

Characteristic	County A	County B	County C	County D	County E	Kenya
Projected Population 2018	1,296,510	158,716	1,033,398	879,694	994,135	45,108,414
Distribution of health facilities by ownership Public	43.7%	67.2%	62.5%	66.3%	65.1%	49.8%
Percentage of County budget allocated to health FY 2017/18	24%	25%	33%	26%	20%	Average percentage of 27%
Per capita allocation to health by county (KES) FY 2017/18	2000	6000	2500	2000	2000	Average of KES 2227

In each county, we selected three public health facilities to represent the different levels of service delivery: one county referral (level 5) hospital, one subcounty hospital (level 4) hospital and one health centre (level 3). Approval to conduct the study in these health facilities was obtained from the different institutional authorities. Table 2 outlines the characteristics of the selected health facilities which are anonymized to maintain confidentiality of participants.

**Table 2:** Characteristics of study health facilities

Characteristic	Hospital 1	Hospital 2	Health centre 1
County	A	A	A
Ownership	Public	Public	Public
Level	Level 4	Level 4	Level 3
Total annual outpatient attendance workload	50,821	91,106	30,367
Total annual inpatient admissions	8960	6713	0
Number of beds	172	183	0
County	B	B	B
Ownership	Public	Public	Public
Level	Level 4	Level 4	Level 3
Total annual outpatient attendance workload	47,171	5964	9150
Total annual inpatient admissions	2856	205	0
Number of beds	305	51	0
County	C	C	C
Ownership	Public	Public	Public
Level	Level 5	Level 4	Level 3
Total annual outpatient attendance workload	248,439	50,084	20,795
Total annual inpatient admissions	5640	1821	30
Number of beds	126	30	1
County	D	D	D
Ownership	Public	Public	Public
Level	Level 5	Level 4	Level 3
Total annual outpatient attendance workload	71,299	51,538	10,813
Total annual inpatient admissions	9964	3035	115
Number of beds	200	89	16
County	E	E	E
Ownership	Public	Public	Public
Level	Level 5	Level 4	Level 3
Total annual outpatient attendance workload	23,338	14,812	11,332
Total annual inpatient admissions	5142	463	221
Number of beds	155	14	11

### Study participants

We purposively selected respondents with knowledge of and experience in health resources and public finance management process which were our study interests. We selected participants at county and health facility level who included county department of health officials and health facility managers and administrators, respectively (Table 3).

**Table 3:** Summary of respondents

County level and health facility respondents	Male	Female	Total
County department of health officials (county director of health, county health administrative officer, county health accountant)	4	1	5
Public hospital managers (Medical superintendent, clinical officer in-charge, nursing office in-charge, hospital administrator)	7	3	10
Hospital accountant	5	–	5
	16	4	20

### Data collection

We collected data between June and August 2019 through in-depth interviews (IDIs) and document reviews. Two researchers (AK and SO) conducted 20 IDIs in English with participants from the county and facilities using semi-structured interview guides developed in reference to the different stages and processes in health resource tracking (See Additional file 1: Semi-structured interview guide). The construct validity of the semi-structured interview guides was tested by health financing experts in our research organization and the collaborating institution in Kenya, to check for ambiguities and leading questions. All IDIs were conducted at the participant’s workplace and were audio-recorded with the participants’ consent using encrypted audio-recorders. Each IDI lasted between 40 and 60 min. Two researchers (AK and SO) held face-to-face peer de-briefing sessions after conducting IDIs to critique the data collection process and identify areas that needed further probing [14]. Finally, we reviewed financial and administrative documents regarding the financial and non-financial resources, health facility expenditures, and grey and peer-reviewed literature on public finance management processes.

### Data management and analysis

The audio recordings from the IDIs were transcribed verbatim in English. All transcripts were compared against their respective audio files for transcription accuracy. The validated transcripts were then imported to NVIVO 10 for coding guided by the topic areas of health resource tracking. The data was analysed using a thematic analysis approach, which involves a process of systematic sifting, sorting, coding and charting data into key issues and themes [15]. One researcher (AK) first familiarized herself with data by reading and re-reading the transcripts. She developed codes from the conceptual framework and applied the codes to segments in the transcripts that were important. Study team members (AK and EB) reviewed and discussed the initial coding framework, and any discrepancies were appropriately reconciled. The final coding framework was applied by (EB and AK) to the data and later charted the data to allow the emergence of themes through comparisons and interpretations. The descriptive analysis of the quantitative data was done in Ms. Excel 2016.

### Ethical considerations

This study received ethics approval from the KEMRI Scientific and Ethics Review Unit (SERU), approval number KEMRI/SERU/CGMR-C/132/3735, Council of Governors, Kenya, National Commission for Science, Technology and Innovation (NACOSTI) serial no. A17531 prior to data collection. Informed consent both written and oral was obtained from potential participants before the interviews were conducted. All study participants were presented with information on the organization conducting the study, who the researchers were, the purpose of the study, the right to withdraw and measures put in place to ensure confidentiality, and gave their written informed consent. Participants were informed that data will be reported in an aggregated format and anonymity will be ensured in storage and publication of the findings of the study.

## Results

### Planning and budgeting

#### Hospital planning and budgeting

The budgeting and planning process and templates for public hospitals varied across the counties. The hospital management teams (HMT), which is a committee that is comprised of the heads of hospital departments, in four of the five study counties (county A, B, C, and E) prepared annual budgets and annual work plans and submitted them to the county department of health. The county departments of health compiled all budgets and plans and integrated them into county plans. However, in one of the counties (county D), hospitals did not prepare budgets and plans, and instead were required to nominate a HMT member to join the county department of health team to directly contribute to the development of an integrated county health department budget and plan. This meant that for this county, public hospitals did not have budgets and plans.

*“Without budgeting you cannot plan properly. And achieving your goals becomes a problem because of the challenges.”* Hospital Accountant, public hospital 2, County D

Further, hospitals in four (county A, B, D, E) of the five study counties did not have visibility of the final budgets allocated to them in the county integrated budgets and felt that the budgets prepared at the hospital were “wishlists” that did not get implemented in practices. This lack of transparency and credibility of the hospital budgeting process made it difficult for them to plan.

*“You cannot budget without an allocation. You must be allowed to access and have visibility of the funds allocated to you to enable you to plan properly.”* Hospital Accountant, public hospital 2, County D

*"We are not privy to how much budget has been allocated to this hospital. I don’t know if they [county department of health] have health specific budgets or they only have one budget for the entire county health department. This detail is not available.”* Medical Superintendent, public hospital 1, County A

However, hospitals in county C received communication and were therefore aware of the budgets allocated to them in the county health departments integrated budgets. This facilitated planning by county C. Hospitals in County C, would present their budgets to a committee that sits in the office of the Chief Officer to present and defend their budgets on a quarterly basis.

#### Health Centres planning and budgeting

Unlike hospitals, the budgeting and planning process for health centers in all the 5 study counties was standardized. Like hospitals, health centers developed annual budgets and plans and submitted them to county departments of health, who integrated them into county department of health budget and plan. However, unlike hospitals, county departments of health communicated back to health centers the financial allocated budgets and, hence, health centers in all 5 study counties had visibility of their final allocated budgets. Budgets at health centres were restricted to allocations from the conditional grants received from donors.

### Sources of funds

#### Hospital source of funds

Table 4 outlines the sources of monetary resources for all health facilities in the study counties. In four of the five study counties (A, B, D, E), hospitals received monetary resources from two main sources, namely user fee collections and reimbursements from NHIF schemes. Hospitals in three of the study counties (A, E, and D) relied heavily on out of pocket payments by patients as a source of financing; user fee collections contributed 78, 60 and 33% of hospital cash resources respectively. Public hospitals in the remaining two counties (B and C) had minimal or no reliance on user fees and instead relied on prepaid financing mechanisms. Specifically, hospitals in county C, which operated a county Universal Health Coverage (UHC) scheme, received most of their cash resources from reimbursements from the county UHC scheme, while hospitals in county B relied on NHIF reimbursements.

**Table 4:** Sources of cash revenues for hospitals and health centres financial year 2017/2018 (KES)

	County A	County B	County C	County D	County E
**Hospital revenue sources**					
**User fees collection**	21,424,561 (78%)	–	2,437,030 (3%)	5,799,125 (33%)	17,156,381 (60%)
**NHIF payments**	5,955,333 (22%)	3,444,586 (100%)	2,684,410 (4%)	11,717,185 (67%)	11,636,383 (40%)
**County financial grants**	–	–	4,800,000 (7%)	–	–
**County health care scheme- reimbursement**	–	–	63,216,980 (86%)	–	–
**Health Centres revenue sources**					
**User fees collection**	1,156,610 (15%)	–	–	–	–
**User fees reimbursement**	–	–	–	–	–
**NHIF payments**	843,709 (11%)	–	–	524,000 (68%)	–
**County financial grants**	–	24,000 (4%)	125,000 (28%)	–	–
**DANIDA**	2,148,882 (27%)	235,332 (35%)	322,425 (72%)	250,000 (32%)	280,000 (100%)
**Financial donor support**	3,705,500 (47%)	408,000 (61%)	–	–	–

#### Health centres source of funds

On paper, public health centres received monetary resources from: 1) conditional grants for user fees reimbursement from the national government 2) operations and management fund supported by a donor (DANIDA), 3) other donor funds, 4) budget allocation from the county (financial grants only in county C), and 5) NHIF reimbursements. In practice, none of the counties received the user fee reimbursement conditional grant from the national government for the financial year 2017/18. They all received funds from the DANIDA program, making them particularly reliant on donor financing. Further, health centers in county A collected user fees, which is contrary to the existing government policy of user removal in primary healthcare facilities. NHIF payments flowed to health centers in only two of the five counties (A and D).

##### Hospitals and health centres experienced delayed and unpredictable funds disbursements

**Fig. 2:**
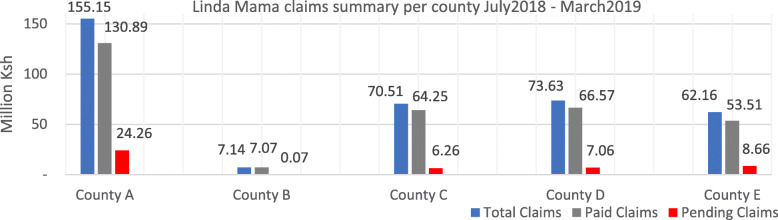
Linda Mama claims summary for July 2018 – March 2019

Funds disbursements to hospitals and health centers from the NHIF was characterized by delays, and unpredictability in terms of amount. The health facilities reported discrepancies between the value of approved claims and the payments received by health facilities for instance, the Linda Mama free maternity scheme (Fig. 2).

*"They [NHIF] don’t pay regularly, and the amounts are not predictable. Currently they [NHIF] owe us a lot of money. They [NHIF] decide what they want to pay us and this decision seems not to be based on how much they owe us." - Hospital Accountant, public hospital 1, County E*

*“We can see the claims that have been submitted, what claims have been processed, and how many have been paid on the NHIF system. However, our experience is that what is reflected on the system is not the same as the money that is transferred to our hospital account. There is a need for reconciliation between the system and the account."* Hospital Accountant, public hospital 1, County A

Similarly, disbursements under the DANIDA program and the user fee reimbursement fund were unpredictable and irregular. This resulted from delayed fund disbursements from the national government.

*"The DANIDA funds are supposed to be disbursed to us quarterly, similar to the user fee forgone reimbursement. We expect this disbursement between January. It is now April and we still haven’t received it." Facility In-charge, public health centre 1, County A*

*" Disbursements are supposed to be quarterly but, for instance this year we have only received two disbursements. In the previous year we only received funds disbursement for one quarter. It is very unpredictable."* Facility In-charge, public health centre 1, County B

### Funding flows

#### Hospital flow of funds

**Fig. 3:**
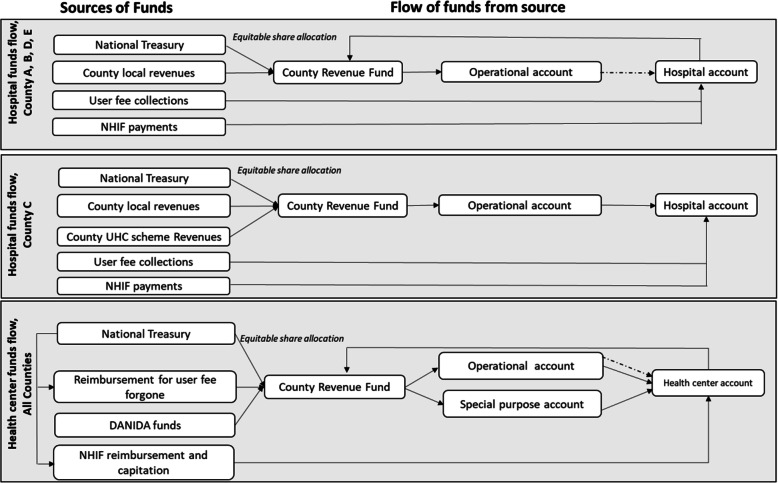
Flow of funds to public hospitals and health centers in the study counties

The flow of funds varied across counties. In one of the study counties (county C), public hospitals retained all cash revenues received in their hospital bank account and hence had access to all their cash revenues (Fig. 3).

However, in four of the study counties (county A, B,D, and E), the hospitals were required to send all funds to the central county revenue fund (CRF), or the funds received in the hospital bank account were immediately redirected to the CRF (Fig. 3). This meant that hospitals in these counties did not have access to cash revenues. Hospitals in one of these counties (county E) retained some of their cash revenues (from NHIF reimbursements) in their hospital bank accounts and hence had access to some but not all their cash resources.

*"NHIF reimburses funds to health facilities, then facilities send these funds to the County Revenue Fund. The county does not send back the money to the facilities. Instead, the county pays directly for health facility expenses."* County Accountant, County D

Hospital bank accounts in these four counties were used only as a transfer mechanism.

*"The NHIF asked us [the hospital] to open an account, but we don't do anything with that account. We just transfer back the money to the county revenue fund. We don't use it. It is only there to receive the funds from the NHIF and to transfer it to the county revenue account."* Hospital Accountant, public hospital 2, County D

The lack of financial autonomy was attributed to the PFM act (2012) that required that all county revenues are remitted to a central account, the CRF. However, it appears that counties had varying interpretations of the PFM act, with county C allowing hospitals to retain financial autonomy without contravening the PFM act.

*“All these monies including donations and conditional grants have to pass through the county revenue fund. It is a legal requirement under public finance management act."* Seconded County Accountant, County A

*“Our hospital collects and retains the money they have collected 100%. But the UHC monies come through the county revenue fund account which we transfer to them. The health centres and dispensaries are given money from the county revenue fund account because they do not collect revenue, apart from Linda Mama. For the rest of the resources, we wire money from the county revenue fund account.”* County Health Administrator, County C

The lack of financial and procurement autonomy had various implications. First, hospitals experienced procurement delays because procurement requests had to be sent to the county health departments which carried out procurement on behalf of hospitals. This affected service delivery.

*“The procurement process is now very lengthy. Procurement requests go to the county department of health, then to the county treasury. This can take one month, two months, three months and it delays a lot of things.”* Medical Superintendent, public hospital 1, County B

*“Every time there is a stock out of a certain commodity. We are operating at bare minimum. There are very essential things that are missing. "* Hospital Accountant, public hospital 1, County A

Second, health facilities had reduced motivation to follow-up on unpaid NHIF claims, which was also due to the time-consuming nature of the process. To address the problems with autonomy, some counties developed county level laws that provided for ringfenced funds for hospitals that allowed hospitals to use funds at source. Of the study counties, 3 out of the 5 counties had developed such a law, 1 county is in the process of developing a law, and 1 county had no law.

#### Health Centre flow of funds

The flow of funds for health centres was standard in all counties (Fig. 3). Conditional grants, and donor funds (DANIDA and other donors) were directly deposited into the county revenue fund (CRF) and then into the health centre accounts through a special purpose account (SPA) of the county department of health. The use of the SPA facilitated the flow of funds directly to health centers, in contrast to hospitals which did not retain funds in their accounts. NHIF reimbursement and off budget donor support was directly deposited into the facility bank account.

The user fees amount reimbursed was based on the health centres workload documents in monthly reports. These were submitted to the county as supporting documents. Health centres were aware of the amounts to be reimbursed.

*"The reimbursement is based on facility workload. Our [facility] workload has kept going up, so the monies are increasing with every reimbursement." Facility In-charge, public health centre 1, County C*

*"The amount received is based on the workload. A facility gets funds based on these services that have been offered."* Facility In-charge, public health centre 1, County A

##### The NHIF reimbursement process was lengthy and time consuming

Health facilities submitted online NHIF claims and tracked them through the NHIF e-claim system. The reimbursements were made in lump sum through bank transfers. However, tracking the reimbursed amounts and rejected claims was a time-consuming and lengthy process, and was complicated by reverting to manual reports.

*" From the reimbursements you cannot tell the difference between the amount for NHIF scheme or Linda Mama scheme. You then must reconcile the claims made against the total amount received. Even after the reconciliation, you might find for example two out of five claims did not meet the threshold for reimbursement." County Accountant, County A*

*"With the E-claim system we can track claim amounts, but we cannot do so with the manual system. We can’t tell whether claims were rejected, or approved, or returned to us. "* Hospital Accountant, public hospital 2, County D

### Health facility expenditure

**Fig. 4:**
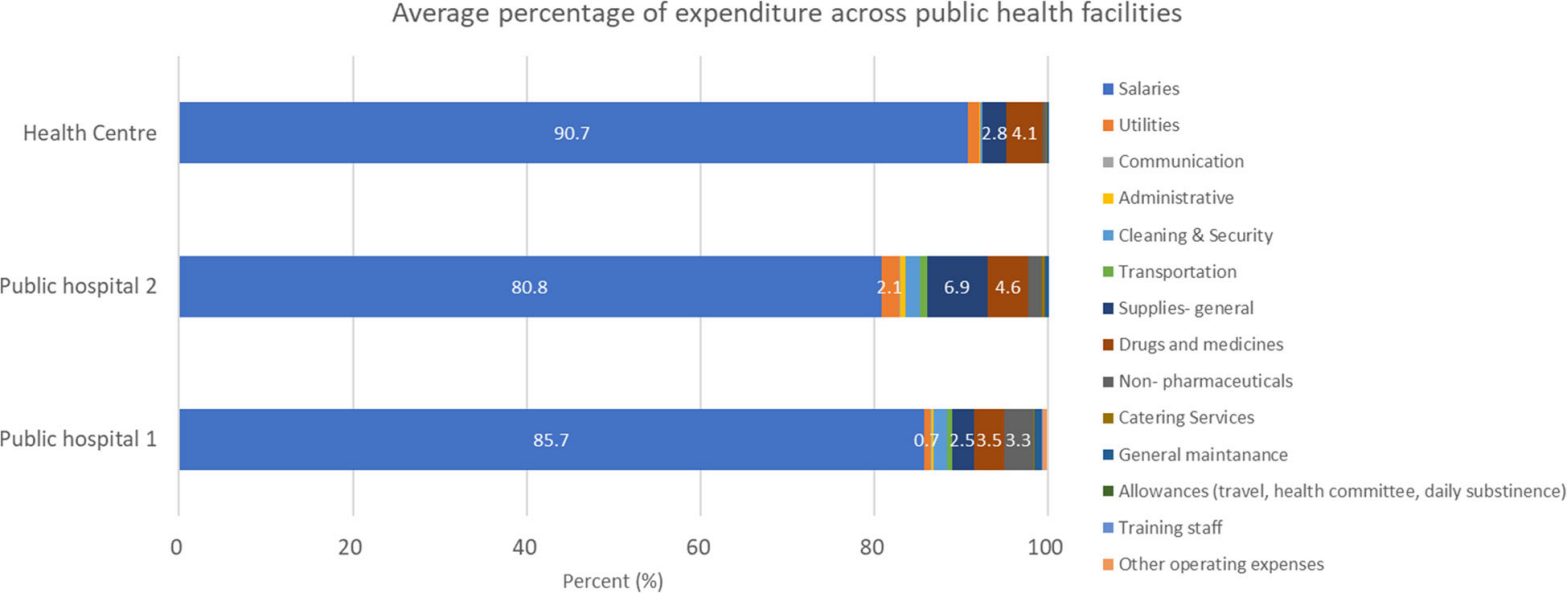
Average percentage of expenditure across the sampled public health facilities

Health facility expenditure was incurred at both health facility and county levels (Table 5). Staff salaries for county employees and contractual staff were paid by the county for both health centres and hospitals. These accounted for 80–90% of the health facility expenditures (Fig. 4).

**Table 5:** Summary of health facility expenditure items

	County A	County B	County C	County D	County E
**County level expenditure**	**Hospitals** all expenditure **Health centre** Salaries, drugs and non-pharmaceuticals, utilities, transport (sometimes)	**Hospitals & health centre** Salaries, drugs & non-pharmaceutical, supplies-general	**Hospitals & health centre** Salaries, equipment purchase, drugs & non-pharmaceutical, maintenance buildings	**Hospitals** all expenditure (except casual labour wages) **Health centre** Salaries, utilities, supplies- general, drugs & non-pharmaceuticals	**Hospitals & health centre** Salaries, utilities, supplies- general, drugs & non-pharmaceuticals
**Facility level expenditure**	**Hospitals** no expenditure **Health center** Supplies- general, casual wages, other costs, communication costs, administrative, cleaning & security, transport, other costs	**Hospitals & health centers** Operations & maintenance, casual labour wages	**Hospitals** Utilities supplies (general), casual wages, administrative, communication, transport, other costs **Health centre** Supplies-general, casual wages, administrative, communication, transport, other costs	**Hospitals** casual labour wages **Health centre** casual wages, maintenance, food, facility developments	**Hospitals & health centre** Casual labour wages, operations & maintenance

Additionally, operation costs, supplies and commodities expenses were paid for at county level for hospitals with no access to funds.

Drugs (3.5–4.6%) and supplies (2.5–6.9%) were the next significant health facility expenditure items. The main source of drugs and supplies was Kenya Medical Supplies Agency (KEMSA). However, in counties with unsettled bills, Mission for Essential Drugs and Supplies (MEDS) was the main supplier and supplemented by local contracted suppliers within the county. On average 70% of drugs and supplies are from KEMSA, and 20 and 10% from MEDS and local suppliers. However, in some facilities KEMSA was the sole supplier.

## Discussion

This study presents an analysis of public health facility financing in Kenya within the context of a devolved health system. Several observations emerge. First, it appears that financing arrangements for public facilities are neither harmonized nor standardized across counties and levels of care (hospitals and health centers). Specifically, budgeting and planning processes, sources of funds, and flow of funds for public hospitals varied across the five study counties but were standardized for health centers across the counties. This fragmentation of financing arrangements is unexpected given that under the devolved system of government, these processes are expected to be guided by the same public finance management law (PFM act 2012). This fragmented approach is symptomatic of the disruption of planning and budgeting processes occasioned by devolution [16, 17]. Under the previous centralized arrangement, health facility planning and budgeting was part of a centralized process that was led by the national MOH and guided by standardized templates, processes, and timelines [18]. After devolution, counties coordinate their planning and budgeting but are expected to do so under the guidance of an overarching legislative and policy framework (the PFM act 2012) [16]. Our findings show that this transition of governance arrangements was not seamless, resulting in fragmented approaches that could compromise accountability. This finding is similar to the early experiences of health sector decentralization in the Philippines, where decentralization disrupted and led to fragmentation of administrative structures and processes including budgeting and planning [19].

Second, the study highlights two concerns with regards to sources of financing for health facilities in the public sector. A key concern is that counties are still reliant on user fee collections as the primary source of financing for public hospitals. This signals that residents in these counties are exposed to out of pocket expenditure that are likely to not only promote inequalities in access to healthcare services, but also compromise financial risk protection and fly in the face of the country’s stated commitment to achieve UHC. The findings however highlight positive examples, with two of the five study counties relying on prepayment health financing rather than user fees as a source of financing. One of these counties is implementing a county level UHC scheme that uses county fund allocation drawn from its equitable share and tax financing from the national level to reimburse health facilities for services provided to its citizens, while the other relies on reimbursements from the country’s public health insurer, the NHIF. These two counties offer examples to other counties on alternative financing pathways to reduce reliance on out of pocket payments as a mechanism for financing public health facilities. Indeed, local level innovation has been identified as one of the pathways to yield the positive effects of decentralization of health systems [19, 20]. Another concern is the over-reliance of donor funds to finance health centers. This is alarming as there is a plan for donors to progressively transition from supporting the Kenyan health sector generally, and primary healthcare facilities specifically. Donor transition is expected to put additional fiscal pressure to LMIC health systems, threatening service delivery, and thus requiring them to device ways replace diminishing donor support with domestic resources [21, 22].

Third, consistent with previous findings [12, 23], funds flow to public health facilities from prepaid sources (county and national government allocations, and NHIF disbursements), are characterized by delays in disbursements. This unpredictability compromised service delivery and quality of services by disrupting the availability of health commodities. Unpredictability in disbursements also compromised health facility planning and may incentivize health facility managers to collect user fees since the later are immediately available to health facilities, with a negative impact on increasing inequalities of financing and access. This means that even as counties think of transitioning from out of pocket payments to a reliance on prepaid financing, it is imperative that the predictability of disbursements is addressed.

Fourth, while health centers appear to have autonomy, public hospitals in four out of five counties do not have managerial and financial autonomy with potential negative impacts on service delivery quality and health facility efficiency. While the problem of autonomy has been identified previously [24], our findings show that there are positive examples of counties providing both managerial autonomy and financial autonomy to public hospitals. While four of the five study counties required that all hospital revenues be sent to a centralized county revenue fund, one of the study counties allowed hospitals to retain their revenues in their hospital account and to spend these funds at the hospital level. Further, all counties allowed health centers to retain funds in their facility accounts and to spend them at this level. While counties in Kenya have cited the public finance management law as the reason for recentralizing financial management away from public hospitals to the county department of health [24], it is clear from these findings that counties can indeed provide financial autonomy to health facilities, the current legislative environment notwithstanding. One solution that has been pursued by counties to resolve the facility autonomy problem is to develop county level laws to allow facilities to retain and manage their funds [25]. However, it is instructive that in one of the study counties that has developed such a bylaw hospitals still do not have financial autonomy, while the one county that has provided financial autonomy to its public hospitals does not have a county level bylaw. This highlights the fact that hospital financial autonomy problem is a political rather than a techno-legal problem and will need to be resolved with this in mind.

Fifth, one of the five study counties, and health centers in all the five counties do not appear to suffer the problem of financial autonomy. The county that does not suffer this problem has attracted attention by being at the forefront of innovation, by introducing a county level UHC plan, doing away with user fees as a source of financing for public hospitals, and guaranteeing managerial and financial autonomy to public health facilities. There is a sense in which the leadership in this county has been progressive and has prioritized the health sector. This perhaps underlies the important role that good leadership and political prioritization plays in supporting positive health system reforms. Further, the fact that health centers have retained their autonomy even as the country transitioned from a centralized to devolved system of governance, highlights the important role that the national government can play to incentivize counties to set up appropriate public finance arrangements. Health centers have retained their autonomy largely because they receive grants from the national government and a development partner (DANIDA) to support their operations and reimburse for user fees forgone. These “extra-county” financial resources are structured as conditional grants that are to be ringfenced for health centers. They also require that health centers have functional health facility management committees that oversee the management of these funds, that health facilities develop budgets and plans, and that they have operational bank accounts. These requirements have clearly been acquiesced to by county governments, arguably because there is a strong financial incentive to do so (the health centers, after all, are owned by county governments). This is perhaps indicative of the potential role that the national government and other national level actors like the NHIF can play in nudging/incentivising county governments to provide autonomy to hospitals in the same way as health centers.

Lastly, findings on health facility expenditures reveal that expenditures on staff salaries dominate. This crowds out expenditures on health commodities and other expenditures needed for effective service delivery and is perhaps indicative of underfunding of healthcare facilities. When health facilities are underfunded, they are likely to spend most of their resources on pre-existing commitments such as staff salaries and neglect other health system inputs such as essential health commodities and thereby compromising efficiency and quality. This seems to be happening in health facilities in the study counties and likely to lead to inefficiency of resource allocation.

## Conclusion

A key limitation of the study is the small number of study counties. This is especially important because PFM practices appear to be varied across counties. This means that the findings from this study cannot be generalized to all the 47 counties. Nonetheless, it is never the intention of qualitative studies to be statistically generalizable but rather to unpack an issue of interest within its context and to be analytically generalizable: the meta issues identified by the study are likely to be found in other counties even though they might manifest in different ways. The limitation notwithstanding, several recommendations for policy can be drawn from our findings.

First, the national government and county governments should consider reviewing the planning and budgeting process for county health systems with a view of developing harmonized and well-coordinated processes. This should be accompanied by comprehensive communication ad awareness creation of this plans at the county level. Second, the national MOH and county governments should put in place mechanisms to transition public health facility financing at the county level away from a reliance of user fees. Given that Kenya is currently scaling up a UHC scheme spearheaded by the national MOH, it is imperative that this scheme is designed such that it replaces user fees as a source of financing for public health facilities with a prepayment mechanism. The current design of the UHC scheme, whereby the government is providing health insurance subsidies for the poor is inadequate in addressing this problem, because a large proportion of the population in need is not covered, the government will need to allocate additional tax revenues to provide coverage for the remaining uncovered population, mostly in the informal sector. While this might be a long-term plan, it appears that county led UHC initiatives might offer short to medium term solutions. Counties should therefore be encouraged and supported to be innovative and develop county level prepayment financing mechanisms financed by county resources (both from allocations from the national government and locally generated revenues). Third, the national government and county governments should also develop and implement plans to facilitate transition of primary healthcare facility financing from donor support. When viewed together with transitioning away from user fees, what is needed by counties is to develop and implement plans to expand their fiscal space for health. Counties will therefore need to review existing and potential sources of financing, with a view to optimize existing sources, and to prospect for additional sources financing. From our findings, it appears that an immediate area to optimize may be resolving challenges with 1) the predictability and timeliness of funds disbursements from the national government and county governments to health facilities and 2) health facility capacity to make claims for reimbursements from the NHIF and the NHIF’s capacity to process claims and make timely disbursements to health facilities. Fourth, there is need for both structural reforms of the PFM laws and political engagement of national and county level political and bureaucratic actors to facilitate financial autonomy of public hospitals. Lastly, county governments should assess health facility resource needs to determine appropriate levels of financing for public health facilities. Such information should then guide appropriate levels resource allocation to public health facilities in ways that facilitate efficient resources allocation and expenditure patterns by public health facilities.

## Supplementary Information

**Additional file 1.**

## Data Availability

The datasets generated and/or analysed during the current study are not publicly available due to participant confidentiality but are available from the corresponding author [AK] on reasonable request.

## Abbreviations

CRF: County Revenue Fund
DANIDA: Danish International Development Agency
EFT: Electronic Funds Transfer
HFC: Health Facility Committee
HFMC: Health Facility Management Committee
HMT: Hospital Management Team
HSSF: Health Sector Services Fund
IDI: In-depth interview
KEMSA: Kenya Medical Supplies Agency
MEDS: Mission for Essential Drugs and Supplies
MOH: Ministry of Health
NHIF: National Hospital Insurance Fund
PFM: Public Finance Management
SPA: Special Purpose Account
