# Supplementary material for: Examining health facility financing in Kenya in the context of devolution
Source: BMC Health Serv Res. 2021 Oct 13;21:1086. doi: 10.1186/s12913-021-07123-7 (PMC8515645; doi:10.1186/s12913-021-07123-7)
Supplement: Supplementary file 1 — Additional file 1. [file 12913_2021_7123_MOESM1_ESM.docx]

**Health Facility Resource Tracking Study**

# Overview

**Purpose**

1. To examine how resources flow through the system to public healthcare facilities in Kenya (hospitals and health centers)
2. To document public financial management practices at the county level

**Primary unit of observation:** Health facilities in the public sector (hospitals and health centers)

**Sample:** To be added

**Data collection method**:

1. Interviews with facility managers (one facility manager per facility]
2. Review of financial records (from the county, and the facility depending on where you get the information)
3. Interviews with County officials (one county official, the one with this information)

**Reference fiscal years**: FY 2017-18

# Key Questions

**COUNTY DEPARTMENT OF HEALTH OFFICIALS QUESTIONNAIRE**

**Planning and budgeting**

1. Could you explain to me how public hospitals, health centers, and dispensaries plan their activities (do they develop work plans)?

*Probe to get specific information about*

*-Public hospitals*

*-Health centers*

*-Dispensaries*

1. Could you explain to me how public hospitals, health centers, and dispensaries budget for their activities?

*Probe to get specific information about*

*-Public hospitals*

*-Health centers*

*-Dispensaries*

1. What role does the county play in the planning and budgeting by public health facilities?

*Probe to get specific information about*

*-Public hospitals*

*-Health centers*

*-Dispensaries*

**Facility financing**

1. What are the sources of financial resources for public hospitals?
2. What are the sources of financial resources for public health centers?
3. What are the sources of funds for public dispensaries?
4. How much came from each of the sources for facility x and y(your sample facilities) for the financial year 2017/18? – ask to be provided with documents to extract this information
5. How else does the county provide resources for public health facilities?

*Probe to get specific information about*

*-Public hospitals*

*-Health centers*

*-Dispensaries*

1. How much did the county spend on staff salaries for facility x and y (sample facilities for the financial year 2017/18? – ask to be provided with documents to extract this information
2. How much did the county spend on pharmaceutical and no-pharmaceuticals for facility x and y (sample facilities for the financial year 2017/18? – ask to be provided with documents to extract this information
3. How much did the county spend on operations and maintenance for facility x and y (sample facilities for the financial year 2017/18? – ask to be provided with documents to extract this information

**Flow of funds**

1. For each of the sources of financial resources for the public hospital/health center/dispensary, describe whether and how the funds get to health facilities
2. Public hospitals/health centers/dispensaries do not receive any financial resources, they are sent to the CRF account
3. Financial resources are sent to the CRF account, and thereafter disbursed to health facilities bank account
4. Financial resources are sent directly to health facilities bank account

*Probe to get specific information about*

*-Public hospitals*

*-Health centers*

*-Dispensaries*

1. Do public hospitals/health centers/dispensaries in your county maintain bank accounts?

*Probe to get specific information about*

*-Public hospitals*

*-Health centers*

*-Dispensaries*

1. Who are the signatories of these facility bank accounts?

*Probe to get specific information about*

*-Public hospitals*

*-Health centers*

*-Dispensaries*

1. Do NHIF funds go directly to public hospitals/health centers/dispensaries bank accounts?

*Probe to get specific information about*

*-Public hospitals*

*-Health centers*

*-Dispensaries*

1. Are public hospitals/health centers/dispensaries allowed to spend these funds or do they have to be sent to the CRF account?

*Probe to get specific information about*

*-Public hospitals*

*-Health centers*

*-Dispensaries*

1. If they have to be sent to the CRF account, do any of these funds go back to the facility?

*Probe to get specific information about*

*-Public hospitals*

*-Health centers*

*-Dispensaries*

1. If yes, what proportion of funds sent to the CRF account are sent back to the public hospitals/health centers/dispensaries bank account?

*Probe to get specific information about*

*-Public hospitals*

*-Health centers*

*-Dispensaries*

1. If yes, what is the procedure for getting funds from the CRF funds back to the public hospitals/health centers/dispensaries?

*Probe to get specific information about*

*-Public hospitals*

*-Health centers*

*-Dispensaries*

**HEALTH FACILITIES – HOSPITALS AND HEALTH CENTERS QUESTIONNAIRE**

1. **Planning and budgeting**
2. Does this facility prepare annual work plans?
3. Does this facility prepare budgets?
4. If yes to A1, could you describe the facility planning process?

*Probe*

-What is the purpose of the planning? What do you plan for?

-Is there a prescribed template for planning?

-Who is involved

-Over what period is planning conducted – annually? Quarterly? etc

1. If yes to A2 could you describe the budgeting process?

*Probe*

-What is the purpose of the budgeting? What kinds of expenditure does the budget cover?

-Is there a prescribed template for budgeting? If yes, can you give us a copy of the template?

-Who is involved in the process?

-Over what period is budgeting conducted – annually? Quarterly? Etc

- Do you submit the budget to the County Department of Health for it to be included in the county budget?

- If yes, does the County DOH tell you how much it finally allocated to your facility?

1. Overall would you say the planning and budgeting process is a useful process for the facility? Why? Why not?

**B) Facility financing**

1. What are the different sources of funding for this facility?
2. Ask the following for each source of funds for 2017/18

|  | **Did you receive any funds from this source in FY 2017-18?** | **If yes, how much for the year? KES** |
| --- | --- | --- |
| User fee collections from patients |  |  |
| NHIF General scheme |  |  |
| NHIF Linda Mama |  |  |
| NHIF Edu Afya Scheme |  |  |
| HSSF/DANIDA |  |  |
| User fee reimbursement |  |  |
| UHC funds [for Isiolo county] |  |  |
| Makueni care funds [For Makueni county] |  |  |
| County financial transfers to the facility |  |  |
| Financial support from donors |  |  |
| Other |  |  |

1. **Flow of funds**
2. Does the facility operate a bank account? How many?
3. Who are the signatories for each of the bank accounts the facility operates?
4. For each of the sources of funds that you have listed above, are the funds deposited to the facility bank account directly? Which account?

|  | **Are the funds deposited directly to the facility account** | **Which account?** |
| --- | --- | --- |
| User fee collections from patients |  |  |
| NHIF General scheme |  |  |
| NHIF Linda Mama |  |  |
| NHIF Edu Afya Scheme |  |  |
| HSSF/DANIDA |  |  |
| User fee reimbursement |  |  |
| UHC funds [for Isiolo county] |  |  |
| Makueni care funds [For Makueni county] |  |  |
| County financial transfers to the facility |  |  |
| Financial support from donors |  |  |
| Other |  |  |

1. If they are not deposited directly, where are the funds sent to?
2. How do you access funds that are not sent directly to your facility bank account?
3. For the funds that are in your account, are you allowed to spend directly from the account?
4. Do you need any permission/approvals to spend these funds? From who?
5. On average, how long does it take for you to get the authorization to spend funds?
   - 1. 1-2 weeks?
     2. 3-4 Weeks
     3. 2-3 months
     4. More than 3 months
6. **RESIDUAL RIGHTS ON NHIF DISBURSEMENTS AND USER FEE COLLECTIONS**
7. Can your facility retain all the funds from user fee collections and/NHIF reimbursements to finance your “operating budget”?
   - 1. We can retain all funds collected by the facility
     2. We remit all funds collected by us to the County Government, and they give us an annual allocation to cover our operating costs
     3. We can retain a portion of the funds collected to cover our operating costs, and remit the rest to the County Government
     4. We have to remit all funds collected by us to the County Government, and they pay for our operating costs directly [skip to 4 below]
8. If (ii) then ask
   - 1. Is the amount you receive from the county government more or less than the revenue you generate and remit to the county government?
        1. More
        2. Less
        3. Same
9. Is the amount you receive from the county government a certain proportion of the revenue you raise? [skip to section E after answering this question]
   - - 1. Yes
       2. No, it is a flat amount regardless of how much revenue the facility generates
10. Can you reconfirm that your facility remits all the funds you receive from NHIF, user fees, etc. to the county government?
11. Does the county operate a cash impress system?
12. How does the county pay for any of your operating costs?
    - 1. We send them a requisition form, and they release the funds to us
      2. We send them an invoice, and they pay for the cost directly
      3. They do not cover any of the operating costs of the facility
13. **NHIF CLAIMS AND REIMBURSEMENT PROCESS**

**NHIF**

1. How do you track how many claims you have submitted for Linda Mama and Edu Afya?

How often does NHIF pay you? Can we see payments received? Is it smooth or does it fluctuate?

1. Is the payment from NHIF broken down by scheme (General scheme, Linda Mama, Edu Afya), or one lump sum?
2. Can you tell us (and show us from your records) how many claims made for Linda Mama and Edu Afya were paid?

**Payments from HSSF/DANIDA [FOR HEALTH CENTERS]**

1. Do you know how much you are supposed to get?
2. How often do you receive the funds?

**User fee reimbursement [FOR HEALTH CENTERS]**

1. Do you know how much you are supposed to get?
2. How often do you receive the funds?
3. Do you need to submit any information to the County Government in order to get the user fee reimbursement?

**MAKUENICARE(MAKUENI)/UHC SCHEME(ISIOLO) [HOSPITALS HEALTH CENTERS]**

If you receive any funds from the county from the *Makuenicare scheme* (IN MAKUENI) or *UHC scheme* (ISIOLO)

1. Do you know how much you are supposed to get?
2. How often do you receive the funds?
3. Do you need to submit any information to the County Government in order to get the user fee reimbursement?
4. **EXPENDITURE [ASK ONLY IF FACILITY IS EITHER ALLOWED TO RETAIN AND SPEND FUNDS, OR RECEIVES FUNDS FROM CRF ACCOUNT TO SPEND]**
5. How much did the facility spend in total for your operating costs in FY 2017-18?
6. How much did you spend on each of the following in FY 2017-18?

| **Expenditure item** | **Costs in KES** |
| --- | --- |
| Casual labor |  |
| Medicines |  |
| None-pharmaceuticals |  |
| Equipment |  |
| Facility maintenance |  |
| Outreach activities |  |
| Other |  |

1. Do you have to report information about how you spent funds to the County Government?
2. **COSTS COVERED DIRECTLY BY THE COUNTY GOVERNMENT**

**Staff salaries**

1. Does the facility know how much the county government is paying for staff salaries and allowances for that facility on an annual basis?
2. What is the total number of staff who worked in the facility in 2017-18, by type?

- Doctors
- Nurses
- ….

**Drugs and supplies**

1. What is your source of drugs and other supplies?
2. How much comes from KEMSA vs other sources?
3. Can you estimate how much was spent on drugs and supplies for your facility in 2017-18?
4. How often do you receive drugs and supplies? KEMSA and non-KEMSA?
5. Do the deliveries arrive at the same time every month/quarter? – KEMSA and non-KEMSA?
6. How often do you experience stock-outs?\
7. What do they do when you experience stock-outs?

**Utilities and facility maintenance**

Can you estimate how much was spent on utilities and facility maintenance for your facility in 2017-18?
